# Kin17 facilitates multiple double-strand break repair pathways that govern B cell class switching

**DOI:** 10.1038/srep37215

**Published:** 2016-11-17

**Authors:** Michael X. Le, Dania Haddad, Alexanda K. Ling, Conglei Li, Clare C. So, Amit Chopra, Rui Hu, Jaime F. Angulo, Jason Moffat, Alberto Martin

**Affiliations:** 1 Department of Immunology, University of Toronto, Medical Sciences Building, Toronto, Ontario, M5S1A8, Canada; 2 Laboratoire de Radio Toxicologie, CEA, Université Paris-Saclay, Arpajon, 91297, France; 3 Donnelly Centre and Banting and Best Department of Medical Research, University of Toronto, Toronto, Ontario, M5S1A8, Canada

## Abstract

Class switch recombination (CSR) in B cells requires the timely repair of DNA double-stranded breaks (DSBs) that result from lesions produced by activation-induced cytidine deaminase (AID). Through a genome-wide RNAi screen, we identified Kin17 as a gene potentially involved in the maintenance of CSR in murine B cells. In this study, we confirm a critical role for Kin17 in CSR independent of AID activity. Furthermore, we make evident that DSBs generated by AID or ionizing radiation require Kin17 for efficient repair and resolution. Our report shows that reduced Kin17 results in an elevated deletion frequency following AID mutational activity in the switch region. In addition, deficiency in Kin17 affects the functionality of multiple DSB repair pathways, namely homologous recombination, non-homologous end-joining, and alternative end-joining. This report demonstrates the importance of Kin17 as a critical factor that acts prior to the repair phase of DSB repair and is of *bona fide* importance for CSR.

B cells undergo class switch recombination (CSR) to replace their immunoglobulin isotype from one class (IgM) to another (IgG, IgE, or IgA). CSR requires the recruitment and activity of activation-induced cytidine deaminase (AID), an enzyme that catalyzes the deamination of deoxycytidines to deoxyuridines within the immunoglobulin switch regions, generating G:U mismatches^1^,^2^,^3^,^4^. The choice of which isotype to switch to is determined by activation and transcription of particular donor and acceptor switch region promoters and the subsequent generation of sterile germline transcripts which facilitate AID access to the DNA^5^. DNA lesions generated by AID are processed by the ubiquitous base excision and mismatch repair pathways to form double-stranded DNA breaks (DSBs) within switch regions^6^,^7^. These DSBs trigger the DNA damage response (DDR), resulting in the activation of the protein kinase ATM^8^, which phosphorylates and mobilizes a range of downstream effector molecules that trigger processes as varied as DNA repair, cell cycle checkpoint activation, metabolism, and cell death^9^. A crucial ATM substrate involved in repair of DSBs is histone variant H2AX, which becomes γH2AX upon phosphorylation^10^ and as γH2AX, recruits terminal effectors 53BP1 and Rif1 proximal to the DSB to promote non-homologous end-joining (NHEJ)^11^,^12^,^13^,^14^. The primary DSB repair pathway active during CSR is NHEJ^15^,^16^, although alternative end joining (A-EJ) also plays a supporting role to repair CSR-induced DSBs^17^,^18^. Ultimately, successful class switching requires the resolution of these AID-induced DSBs in G1 phase of the cell cycle^19^.

Kin17 (hereafter Kin) was originally identified in murine cells on the basis of robust cross-reactivity to antibodies raised against *E. coli* RecA, a protein involved in DNA repair and recombination in bacteria^20^,^21^. Kin is a ubiquitously expressed and evolutionarily conserved protein^22^ that has been linked to numerous cellular processes, including DNA replication^23^, cell cycle regulation^24^,^25^,^26^ and the response to UV or ionizing radiation induced DNA damage^27^,^28^. In response to UV induced damage, Kin expression has been shown to be upregulated in a manner dependent on the global genome nucleotide excision repair factors XPA and XPC^27^. Furthermore, Kin has also been proposed to function in the response to ionizing radiation^25^,^28^. However, multiple repair pathways – including homologous recombination (HR), NHEJ, and A-EJ – are involved in repair of DSBs generated from ionizing radiation^29^ and whether Kin is specifically involved in the functionality of these processes remains unknown. Furthermore, it remains an open question as to whether B cells require Kin function to repair the programmed DSBs generated during CSR.

Although great strides have been made to advance our understanding of how programmed DSBs generated during CSR are repaired, significant knowledge gaps still remain – especially with respect to DNA damage responses that may be independent of well-studied orchestrators such as ATM or DNAPK. We performed a whole genome loss-of-function screen to identify factors that contribute to CSR^30^. From this screen, we identified Kin as one of the candidate genes involved in CSR. Our results demonstrate that Kin is required for repair of DSBs generated incidentally, as in the case of ionizing radiation, or in a programmed fashion, such as during CSR.

## Results

### Kin is a factor required for optimal CSR

**Figure 1:**
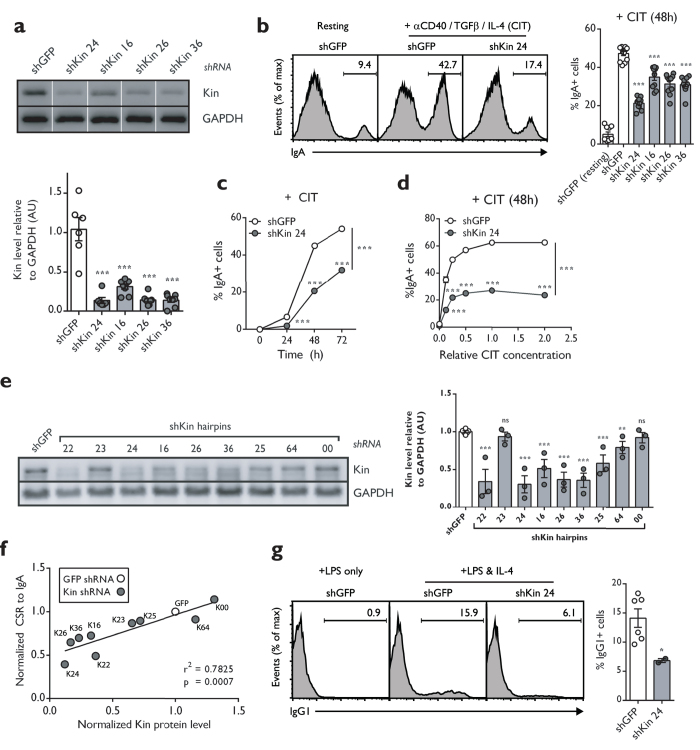
Kin is required for optimal CSR *in vitro* and *ex vivo*. CH12 cells transduced and selected with shRNA targeting negative control GFP (shGFP) and Kin (shKin 24, 16, 26, 36) were measured for (**a**) Kin expression relative to GAPDH by Western blot and (**b**) surface IgA expression following 48 hour CIT-stimulation. Representative plots (left) and summary graph (right) shown. Each shRNA was tested with 2 biological replicates in 3–6 independent experiments. Surface IgA expression in shGFP and shKin 24 transduced CH12 cells (**c**) 24, 48, and 72 hours following CIT-stimulation and (**d**) 48 hours following various doses of CIT-stimulation. Data was analyzed by two-way ANOVA. (**e**) Expression levels of Kin relative to GAPDH as measured by Western blot for CH12 cells transduced with the shRNA described in (**a**) in addition to Kin specific shRNA (shKin 23, 25, 64, 00). (**f**) Correlation of normalized CSR to IgA versus normalized Kin expression following treatment with shRNA shown in (**e**). Data was analyzed by goodness of fit following linear regression analysis. (**g**) Surface IgG1 expression in *ex vivo* mouse splenic B cells transduced and selected with shGFP and shKin 24 and stimulated with LPS & IL-4. Unless otherwise indicated, all experiments were analyzed by unpaired, two-tailed, Student’s t-test.

To identify novel factors involved in CSR, a previously developed shRNA library^31^ was introduced in bulk into the mouse B cell line, CH12F3-2 (hereafter CH12), which is capable of undergoing robust CSR from IgM to IgA *in vitro* upon stimulation with a cocktail composed of anti-CD40, IL-4 and TGFβ (hereafter CIT)^32^. Two Kin-specific hairpins, classified as shKin 22 and shKin 24, were two of the top ranked shRNAs identified from this screen. However, the two hairpins shared considerable sequence overlap (identical except for one nucleotide) and we henceforth treated them as effectively a single shRNA (shKin 24) (see Supplementary Figure S1a and Table S2). In order to rule out potential off-target effects of the shKin 24 hairpin, we designed additional hairpins (shKin 16, 26, 36) and acquired other commercial hairpins that target Kin (shKin 23, 25, 64, 00). These hairpins target different regions within the *Kin* gene (Supplementary Figure S1a). The shKin24 hairpin was effective at reducing both Kin transcript and protein expression (Fig. 1a, S1b) as were multiple additional hairpins targeting Kin that also demonstrated comparable knockdown at the protein level, relative to a negative control shRNA targeting GFP (shGFP) (Fig. 1a). Notably, the same shRNA targeting Kin reduced CSR frequency to IgA in CH12 cells stimulated with the CIT cocktail for 48 hours (Fig. 1b). Knockdown of Kin resulted in 2-fold reduced CSR frequency 24, 48, and 72 hours following CIT stimulation (Fig. 1c) as well as impaired CSR irrespective of the CIT cocktail dosage – ranging from 0.125x to 2.0x the standard concentration of 2 μg/mL anti-CD40, 10 ng/mL IL-4, and 1 ng/mL TGFβ (Fig. 1d). We next examined our large panel of Kin shRNA – revealing that some hairpins were only capable of mild to no knockdown of the protein – with the goal of examining the relationship between Kin expression and the efficiency of CSR (Supplementary Figure S1a, Fig. 1e). To this end, we discovered a striking correlation between the effectiveness of Kin knockdown and the degree of CSR impairment between numerous Kin specific shRNA that were tested (Fig. 1f). Individually, the majority of the shRNA that impaired Kin expression equivalently reduced CSR following CIT stimulation (Supplementary Figure S1c), albeit milder in effect when compared to the efficacy and consistency of shKin 24 (Supplementary Figure S1d). As such, the majority of subsequent experiments in the study were performed with the shKin 24 hairpin. Importantly, Kin knockdown by shKin 24 in *ex vivo* splenic mouse B cells also resulted in a 60% reduction in CSR – in this case, to IgG1 – upon stimulation with LPS & IL-4 (Fig. 1g). These data support the notion that Kin is required for CSR both *in vitro* and *ex vivo*.

We also attempted to generate a Kin^−/−^ CH12 cell line via targeted gene deletion with the CRISPR/Cas9 system, by transfecting WT CH12 cells with Cas9 expressing vectors coupled to Kin-targeting guide RNAs. Over multiple rounds of screening, we were only able to isolate clones bearing heterozygote mutations in the *Kin* gene; on no occasions did we produce homozygous knockouts (data not shown). It is likely that a Kin^−/−^ cell line or mouse may be inviable, an unsurprising conclusion in light of evidence from previous studies^33^. Furthermore, a recent genome-wide CRISPR-Cas9 screen for fitness genes revealed that attempts to knock out Kin in five different human cell lines resulted in lethality in all five cases^34^. Given the pleiotropic involvement of Kin in multiple cellular processes, we believe that the level of knockdown achieved by shKin 24 represents an attainable floor of expression prior to cell inviability.

### Kin facilitates CSR independently of AID

**Figure 2:**
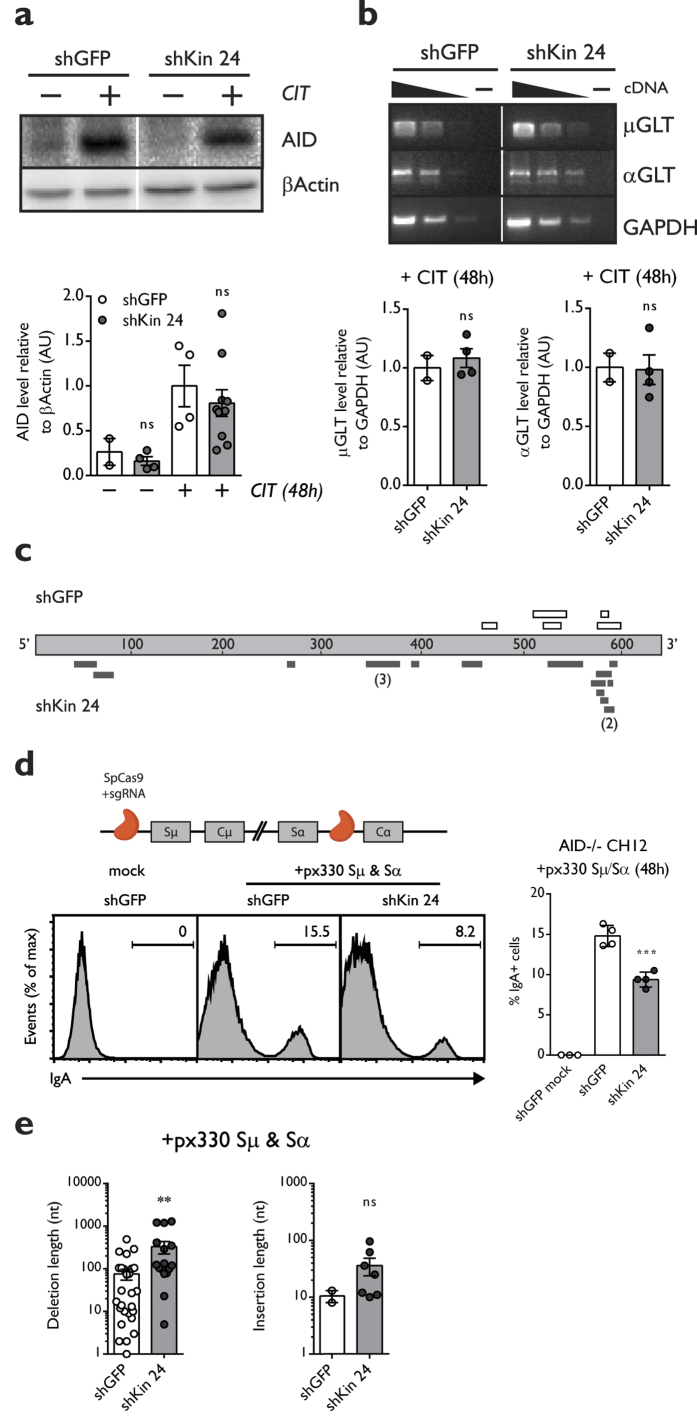
The impact of Kin deficiency on CSR is AID independent. Expression levels of (**a**) AID relative to β Actin measured by Western blot or (**b**) spliced germline μ and α switch region transcripts relative to GAPDH measured by RT-PCR with or without 48 hours of CIT-stimulation in CH12 cells transduced and selected with shGFP and shKin 24. Each shRNA was tested in 2–4 biological replicates in 2 independent experiments. (**c**) Schematic detailing the distribution of unique deletions in the 5′ switch μ region: white (shGFP) and dark gray (shKin 24) bars represent unique deletions according to position and length. Numbers in brackets represent the frequency of a particular deletion in the set. (**d**) Cas9 was targeted to sequences flanking immediately upstream and downstream of the μ and α switch regions to induce CSR via the CRISPR/Cas9 system. Surface IgA expression was measured in AID^−/−^ CH12 cells transduced and selected for shGFP and shKin 24, then transfected with the Cas9 expression vector (px330) equipped with upstream μ and downstream α guide RNAs for 48 hours. (**e**) Junctions from CRISPR/Cas9 mediated CSR events in shGFP and shKin 24 AID^−/−^ CH12 cells were sequenced and analyzed for deletion (left) and insertion length (right). For the above experiments, each shRNA was tested with at least 2 biological replicates in 2 independent experiments. All experiments were analyzed by unpaired, two-tailed, Student’s t-test.

In order to determine if the Kin-specific CSR defect was upstream or downstream of AID activity, we measured AID expression and function in the context of Kin deficiency. We observed that knockdown of Kin did not affect AID levels (Fig. 2a) or expression of germline transcripts at either the μ or α switch regions (Fig. 2b) in CH12 cells 48 hours following CIT stimulation. Furthermore, we sequenced a section 5′ to the μ switch region in CH12 harbouring either shGFP or shKin 24, which revealed a comparable mutation frequency in shKin 24 CH12 cells compared to the shGFP counterpart, indicating that reduced Kin expression did not affect AID activity (Table 1). Despite a similar mutation frequency at the μ switch region, shKin 24 cells harboured a greater frequency of deletions versus shGFP control when compared across the same region (Table 1, Fig. 2c). Notably, no mutations or deletions were present in sequences derived from AID^−/−^ CH12 cells (Table 1).

We also sought to determine whether Kin deficiency would impact CSR in an AID-independent system of class switching. In this case, AID^−/−^ CH12 cells (gift from Kefei Yu)^30^ were induced to switch from IgM to IgA with the CRISPR/Cas9 system by targeting the Cas9 endonuclease with guide RNAs that bind upstream and downstream of the μ and α switch regions, respectively (Fig. 2d, top). Knockdown by shKin 24 resulted in a 40% reduction in CRISPR/Cas9-mediated CSR relative to the control hairpin when measured 48 hours following transfection of the Cas9 nuclease and S region guides (Fig. 2d, bottom).

We then sequenced switch junctions from these shGFP and shKin 24 transduced AID^−/−^ CH12 cells following CRISPR/Cas9-mediated CSR – comprised of resolved DSBs which were generated at a fixed position due to precise targeting by guide RNAs. Analysis of these junction break points revealed substantially larger deletions in shKin 24 knockdown AID^−/−^ CH12 cells when compared to shGFP controls as well as a greater proportion of sequences containing insertions (Table 2, Fig. 2e). These data imply that DSBs undergo differential processing and repair when confronted with reduced Kin activity and also suggest that the CSR defect observed in Kin knockdown cells is independent and downstream of AID function.

### Kin deficiency results in cell cycle arrest upon stimulation or DNA damage

**Figure 3:**
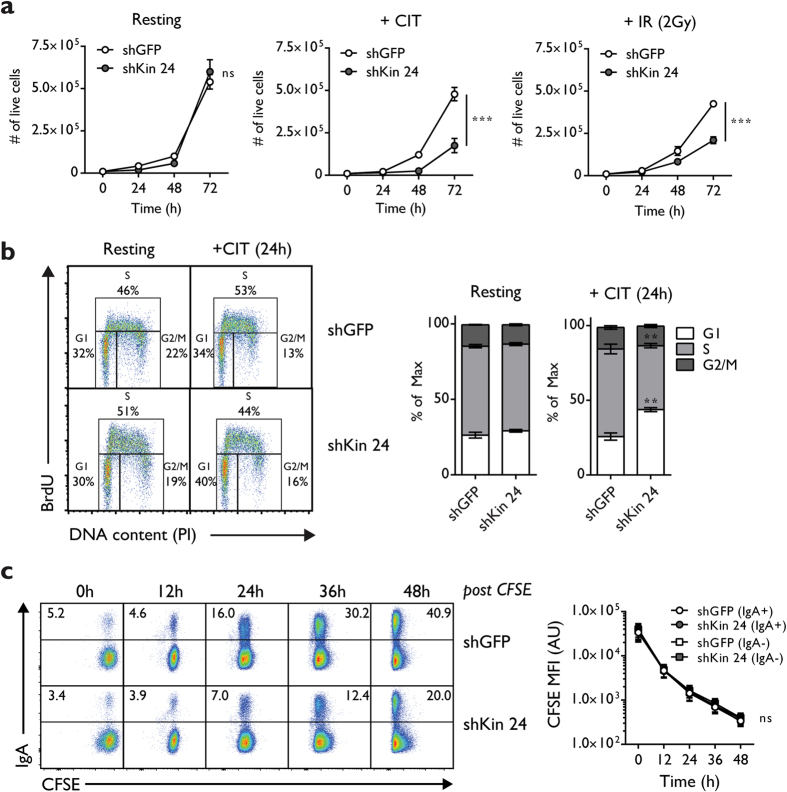
Stimulation or DNA damage results in cell-cycle arrest in Kin-deficient cells. (**a**) Growth curves of CH12 cells transduced and selected with shGFP and shKin 24, while resting, CIT stimulated, and irradiated (2 Grays) over a 72 hour timecourse as measured by Trypan Blue exclusion counting. Data was analyzed by two-way ANOVA. (**b**) Cell cycle profiling of CH12 cells transduced and selected with shGFP and shKin 24 by BrDU/PI flow cytometry staining following 24 hours of CIT stimulation. Representative plots (left) and summary graphs (right) are shown. Each shRNA was tested with 2 biological replicates in 2 independent experiments. (**c**) CH12 cells transduced and selected with shGFP and shKin 24 were pulsed with CFSE, CIT-stimulated, and acquired at various time points following treatment. Representative plots (left) and summary of median fluorescence intensities (MFI) of IgA-positive and IgA-negative cells from each shRNA population (right) are shown. Each shRNA was tested with 2 biological replicates in 2 independent experiments. Data was analyzed by two-way ANOVA. Unless otherwise indicated, all experiments were analyzed by unpaired, two-tailed, Student’s t-test.

Previous studies on Kin have reported an increased sensitivity to DNA damage induced by ionizing radiation in Kin-deficient cell lines^28^,^33^. To test whether Kin-deficient CH12 cells were similarly sensitive to DNA damage, we measured proliferation in Kin-deficient CH12 with by enumerating cells treated with CIT stimulation or 2 Grays of ionizing radiation for 72 hours. We observed reduced rates of proliferation in shKin 24 transduced CH12 in response to CIT stimulation or irradiation induced DNA damage, but no observable defects in resting cells relative to control (Fig. 3a). Equivalent assays on CH12 cells transduced with shKin 16 and shKin 26 recapitulated this proliferative defect to a moderate degree (Supplementary Figure S2a). This defect in cell proliferation in Kin-deficient cells was likely not due to mildly elevated levels of apoptosis as quantified by Annexin V-PI staining (Supplementary Figure S2b) nor a defective mitotic index as measured by phospho-histone H3 levels (Supplementary Figure S2c). From cell cycle distribution analysis with BrdU-PI staining, we discovered that the proliferation defect in CIT-stimulated, Kin-deficient CH12 is partially attributed to arrest in the G1-phase of the cell cycle, a phase which accumulated a greater proportion of Kin-deficient cells (Fig. 3b). Ultimately, these results reflect the notion that DSBs generated by AID during G1 phase^10^ are not readily repaired by Kin-deficient cells, as evidenced by the accumulation of these cells in G1 phase.

As CSR is closely linked with cell proliferation^35^, we evaluated whether the CSR defect in Kin-deficient cells was due to a defect in proliferation in response to CIT stimulation or DNA damage. To achieve this, we devised a timecourse to measure CSR frequency 0, 12, 24, 36, 48 hours following CIT stimulation in conjunction with CFSE labeling to measure proliferation dynamics at each stage (Fig. 3c). Although we observed a constant two-fold decrease in CSR efficiency in shKin 24 knockdown cells at every time point (Fig. 3c, left), we did not detect a significant difference in proliferation as measured by CFSE median fluorescence intensity (MFI) in either the class-switched IgA-positive or the unswitched IgA-negative population in both shKin 24 and shGFP control cells (Fig. 3c, right). These data suggest that the CSR defect in Kin-deficient cells is not due to a difference in proliferation rates between IgA-positive cells in CH12 cells harbouring the shKin 24 hairpin compared to the shGFP hairpin.

### The γH2AX mediated DNA damage response is not affected by Kin knockdown

**Figure 4:**
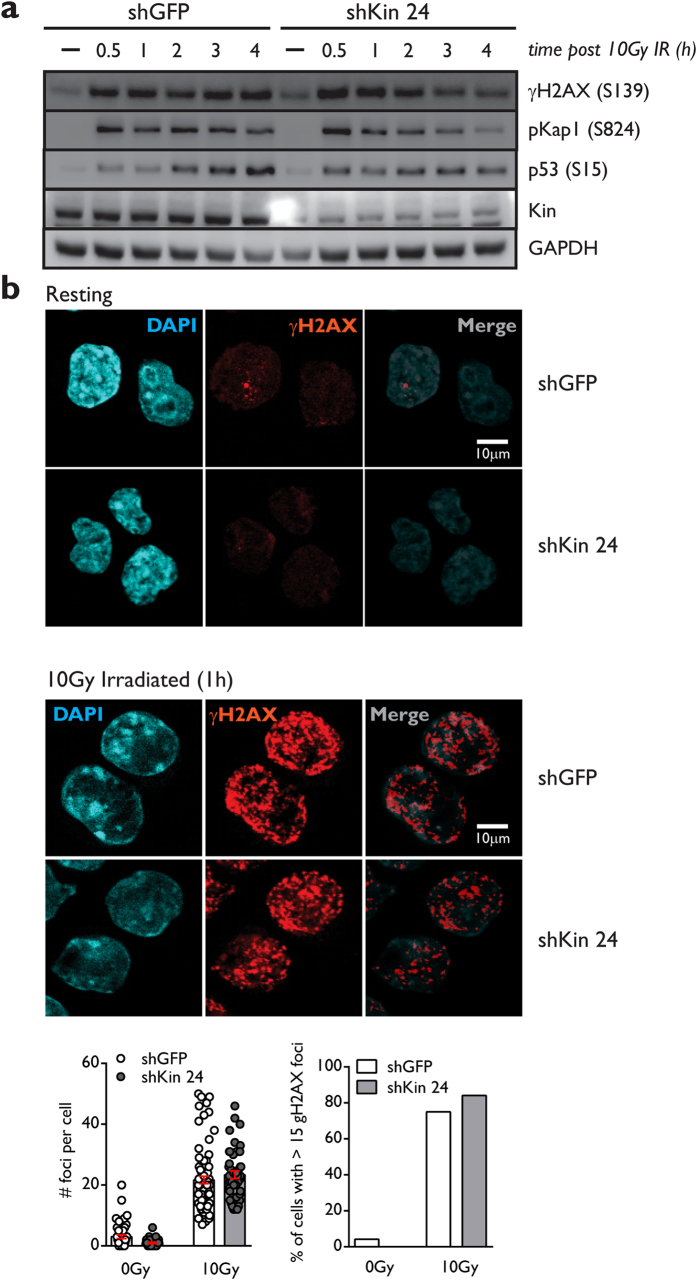
Deficiency in Kin does not impair the γH2AX-mediated DNA damage response. (**a**) CH12 cells transduced and selected with shGFP and shKin 24 were exposed to 10 Grays of ionizing radiation and collected at various time points post-irradiation for Western blot analysis of γH2AX (Ser139), phospho-Kap1 (Ser824), phospho-p53 (Ser 15), and Kin, all relative to GAPDH. Data is representative of 5 independent experiments. (**b**) CH12 cells were transduced with shRNA and irradiated as in (**a**) and collected onto slides via cytospin one hour following irradiation. Slides were subject to immunofluorescence staining of γH2AX and analyzed for number of γH2AX foci per cell. Representative fields of view (top), summary of γH2AX foci per cell (bottom left), and summary of cells with greater than 15 γH2AX foci (bottom right) are shown. Scale bars (top right panels) represent a distance of 10 μm. Data is representative of 2 independent experiments. All experiments were analyzed by unpaired, two-tailed, Student’s t-test, where applicable.

The ATM and DNAPK mediated γH2AX cascade is amplified rapidly upon DSB generation by AID or other forms of DNA damage and is crucial for completion of DSB repair and CSR^36^,^37^. Kap1 Ser824 and p53 Ser15 are primary ATM targets that are phosphorylated as a response to DSB formation in the cell^38^,^39^. Since Kin-deficient CH12 cells demonstrated delayed cell-cycle kinetics in the presence of AID or ionizing irradiation induced DNA damage, we asked whether the early stages of the DNA damage response were disturbed in Kin-deficient cells under genotoxic stress. We detected no observable difference between the kinetics of γH2AX, Kap1 Ser824, or p53 Ser15 phosphorylation at various time points between shGFP or shKin 24 transduced CH12 cells following either 2 (Supplementary Figure S2d) or 10 Grays of ionizing radiation (Fig. 4a). Furthermore, formation of ionizing-radiation induced γH2AX foci – a characteristic of the response to DSB generation – one hour following 10 Grays of exposure appeared phenotypically normal with respect to foci number and proportion of cells with greater than 15 foci per cell in Kin-deficient CH12 cells relative to control (Fig. 4b). Together, these results suggest that the CSR and cell cycling defects in response to DNA damage in Kin-deficient cells manifest subsequent to the recognition of DSBs and initiation of the DDR.

### Kin facilitates multiple avenues of DSB repair

**Figure 5:**
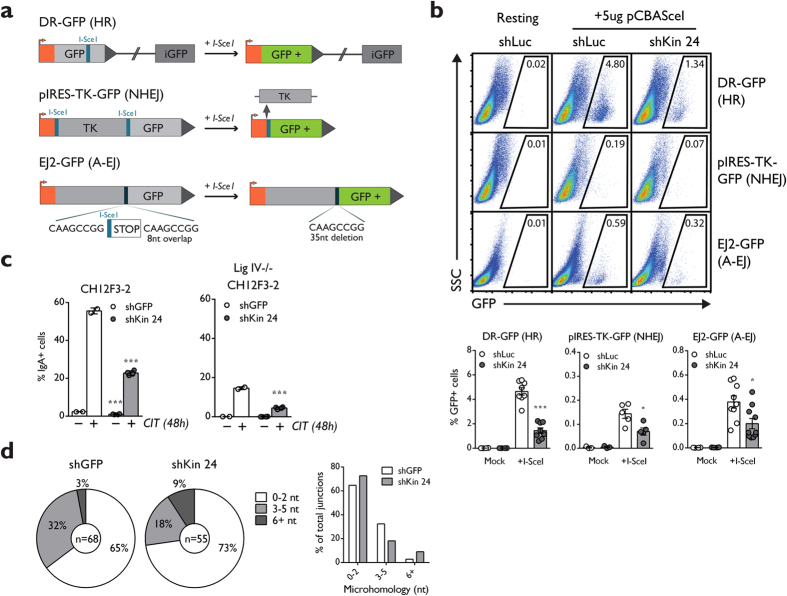
Kin is required for the function of multiple pathways of DSB repair. (**a**) Schematic of repair substrates used in the study. The DR-GFP (HR), pIRES-TK-GFP (NHEJ), and EJ2-GFP (A-EJ) substrates incur DSBs upon introduction of yeast endonuclease I-SceI. These DSBs are repaired by the specified pathway with the use of auxiliary cassettes, and then quantified through GFP fluorescence via flow cytometry. (**b**) CH12 clones harbouring the above substrates were transduced with negative control shLuc and shKin 24 and mock transfected or transfected with I-SceI expressing pCBASceI and acquired by flow cytometry to quantify GFP expression. Representative plots (top) and summary graphs (bottom) are shown. Each shRNA was assayed with 2 biological replicates in 4 independent experiments. (**c**) Surface IgA expression measured in 48 hour CIT-stimulated CH12 or Ligase IV^−/−^ CH12 cells transduced and selected with shGFP and shKin 24. (**d**) Quantification of microhomology lengths in Sμ/Sα junctions cloned from IgA-switched CH12 cells transduced with shGFP or shKin 24. Junctions were classified according to length: near-blunt (0–2 nt, white) or with microhomology (3–5 nt, light gray; 6+ nt, dark gray) and represented in pie chart (left) or bar graph (right) format. Unless otherwise indicated, all experiments were analyzed by unpaired, two-tailed, Student’s t-test.

The presence of an intact initial DDR suggests that the defective CSR phenotype in Kin-deficient cells could be attributed to defects during the subsequent DSB resolution phase. Indeed, previous studies have suggested that Kin could be involved in repair of DNA lesions ranging from irradiation induced DSBs and pyrimidine dimers^27^,^33^. To expand on this notion, we adapted three DSB repair substrates, DR-GFP, pIRES-TK-GFP, and EJ2-GFP to our CH12 system to develop a means to measure homologous recombination (HR), non-homologous end-joining (NHEJ), and alternative end-joining (A-EJ), respectively in these cells^40^,^41^,^42^. Each construct was designed with one or two I-SceI endonuclease sites which can be triggered to create DSBs that can be preferentially repaired by one of the HR, NHEJ, or A-EJ pathways depending on the design of the auxiliary cassettes in each construct, resulting in GFP expression upon repair (Fig. 5a).

CH12 clones harbouring the HR, NHEJ, and A-EJ substrates were transduced with lentivirus harbouring shKin 24 and a negative control hairpin targeting luciferase (shLuc), selected, and then assayed for GFP expression by flow cytometry following transfection of the I-SceI expression plasmid. Knockdown by shKin24 resulted in a 50% or greater reduction in GFP expression in all three DSB repair pathways (Fig. 5b). Parallel analyses on the HR, NHEJ, and A-EJ clones with the additional shRNA mentioned in Fig. 1 (shKin 26, 36) demonstrated reduced GFP expression largely correlating to the extent of knockdown achieved by these hairpins (Supplementary Figure S3). These data confirm that disruption of Kin negatively impacts the function of three separate mechanisms of DSB repair: HR, NHEJ, and A-EJ.

Defective repair in this case could either be explained by a specific involvement of Kin in each pathway or suggest a function for Kin that is upstream of all three pathways of DSB repair. To clarify this situation, we also transduced a previously established CH12 cell line harbouring an inactivating deletion of Ligase IV (Lig IV^−/−^ CH12), an essential component of the NHEJ pathway^15^, with hairpins targeting Kin and GFP. As expected, following 48 hours of CIT stimulation, Lig IV^−/−^ CH12 switch to IgA with a considerably reduced frequency compared to WT CH12 (15% versus 55% IgA-positive cells) (Fig. 5c), with all CSR events in these cells being carried out by the A-EJ pathway in the absence of functional NHEJ. When knocked down for Kin, Lig IV^−/−^ CH12 demonstrate an even further reduction in CSR (Fig. 5c), a defect of comparable proportions relative to WT CH12. To identify whether this defect in Lig IV^−/−^ CH12 cells was a result of a defect specific to A-EJ, we cloned and sequenced μ-α switch junctions in IgA-positive, switched CH12 from WT CH12 transduced with shKin 24 and shGFP. Defects in A-EJ and NHEJ specific factors present with abnormal microhomology usage at the switch junctions, with NHEJ favouring overlap between blunt and 3 nucleotides and A-EJ favouring greater than 3 nucleotides of microhomology^43^,^44^. We observed no differential microhomology usage in Kin-deficient cells with respect to the distribution of blunt (0 to 2 nucleotides) and microhomologous (greater than 3 nucleotides) joins when compared to control (Fig. 5d). Taken together with the evidence that Kin deficiency impacts negatively upon multiple DSB repair pathways, we conclude that Kin’s function lies upstream of DSB repair pathway choice and is not specific to an individual pathway.

## Discussion

Previous studies have linked Kin to multiple avenues of DNA repair, with the protein showing responsiveness to nucleotide excision repair activity^27^ and the formation of irradiation induced DSBs^28^,^33^, yet its biological function remains elusive. In this study, we have taken strides towards understanding the function of Kin by revealing a critical role for the protein in CSR which may reflect its involvement in DNA repair activity upstream of DSB repair pathway choice.

We demonstrate that Kin deficiency impedes the progression of CSR independently of AID, as Kin knockdown cells exhibit normal AID expression and function and maintain reduced CRISPR/Cas9 mediated, AID-independent CSR (Fig. 2). Intriguingly, cells deficient for Kin possessed a notably greater frequency of deletions in the μ switch region (Table 1, Fig. 2c). Deletions of greater lengths were also found at the switch junctions of Kin knockdown cells following CRISPR/Cas9 mediated CSR (Table 2, Fig. 2e). These results are consistent with a defect in DSB repair, as persistent genomic DSBs will be subjected to further processing by exonucleases such as Exo1 and CtIP^45^. Deletions of increased length and frequency in the switch regions may also represent the aftermath of intra switch recombination events, a common outcome of DSB repair in switch regions that has no effect on antibody class^46^. Whether this speaks to a role for Kin in maintaining the physiological frequency of intra- versus inter-switch region recombination, as in CSR, will be the objective of future studies.

Kin knockdown resulted in a cell cycle block at the G1/S boundary following AID induction (Fig. 3b), an enzyme which generates DNA lesions that are repaired in G1 phase^19^. This G1 cell cycle block is indicative of defective repair of CSR generated DSBs, a notion that is supported by the inefficient repair of programmed DSBs generated by yeast endonuclease I-SceI in Kin-deficient cells harbouring constructs that measure HR, NHEJ, and A-EJ activity (Fig. 5b, S3b). Taken together, this suggests that Kin impacts on DNA repair upstream of the decision of pathway choice and earlier in the response to DSB formation.

Recent studies have revealed insights on the post-translational regulation of Kin, specifically through trimethylation of Lysine-135 in the winged-helix domain^47^,^48^. Specifically, METTL22 has been identified as a methyltransferase that contributes to this trimethylation modification, a mark that, when accumulated, leads to dissociation of Kin from chromatin^48^ and could therefore have a major impact on the DNA repair function of Kin in the nucleus. Notably, histone methylation has also been shown to play an important role in marking S regions prior to recombination and facilitating the recruitment and targeting of AID by 14-3-3 adaptors^49^. The role of post-translational modifications of Kin, especially this novel trimethylation mark on Lysine-135, should be subject to further study in the context of the newly developed role of Kin in DSB repair and CSR that we present in this report.

We have demonstrated that Kin plays a prospective role in promoting efficient repair of DSB breaks which is of fundamental importance for the completion of CSR. These observations open up new avenues for the interrogation of this poorly-defined factor and the ultimately, the elucidation of its molecular functions.

## Methods

### Antibodies and plasmids

Antibodies targeting phospho-histone H2AX (γH2AX) (Millipore and Cell Signaling), TIF1β-pS824 (Kap1) (Cell Signaling), p53-pS15 (Cell Signaling), AID (Cell Signaling), Kin17 clone K58 (Santa Cruz), β actin (Sigma), GAPDH (Life Technologies), anti-mouse IgG1 Alexa Fluor 488 (Life Technologies), and anti-mouse Ig H+L Alexa Fluor 555 (Life Technologies) were used as specified by the manufacturers’ protocols for Western blot or Immunofluorescence analysis. The shRNA plasmids used in this study include pLKO.1-puro (The RNAi Consortium) and pLKO.005_TRC018-hygro (Gift from Jason Moffat). The CRISPR/Cas9 RNA expression plasmid px330 was use for CRISPR-mediated CSR assays (Zhang Lab, Addgene). DNA repair substrates included DR-GFP (Jasin Lab, Addgene), pIRES-TK-GFP (Gift from Takashi Kohno), and EJ2-GFP (Stark Lab, Addgene).

### Cell culture and *in vitro* assays

CH12F3-2 (CH12) lymphoma B cells, obtained from Dr. Tasuku Honjo, were cultured and CSR assays were performed as described previously^50^. CH12 were stimulated with 1 ng/mL recombinant human TGFβ1 (R&D Systems), 10 ng/mL recombinant mouse IL-4 (R&D Systems) and 2 μg/mL functional grade purified anti-mouse CD40 (eBioscience) for various time points, and analyzed by flow cytometry, as described below. For growth curve analysis, CH12 cells were diluted to a fixed concentration and aliquoted into 96-well plates. The numbers of trypan blue excluded cells were enumerated with a haemocytometer at various time points. AID^−/−^ and Ligase IV^−/−^ CH12 cells were generated as described previously^15^. For DSB repair studies, CH12 were irradiated with various doses of ionizing radiation using a Nordion Gammacell 1000 irradiator at a rate of 1 Gray/9.75 seconds. All experimental protocols were approved by the University of Toronto Biosafety program under certificate 199-M09-2.

### Lentiviral shRNA transduction

Negative control lentiviral shRNA constructs used in this study include shGFP (TRCN0000072181) and shLuc (TRCN0000072243) acquired from The RNAi Consortium. shRNA constructs targeting Kin include shKin 24 (TRCN0000103924) and shKin 22 (TRCN0000103922) acquired from The RNAi Consortium; shKin 16, 26, 36 cloned *de novo*; and shKin 23, 25, 64, 00 purchased from the MISSION shRNA library (Sigma-Aldrich). CH12 cells were transduced with lentivirus generated from HEK293T cells for 24 hours, on 24 well plates for bulk transductions or diluted and plated on 96 well plates to obtain clones. Positively transduced cells were selected with 1 μg/mL puromycin (Wisent Bio Products) for 3–4 days or 2 mg/mL hygromycin (Wisent Bio Products) for 6–8 days.

### RT-PCR analysis

RNA was extracted from CH12 cells with TRIzol (Life Technologies), followed by DNaseI treatment (Fermentas) and reverse transcription with either Superscript III (Life Technologies) or Maxima (Thermo Scientific) to prepare cDNA. Samples were titrated and subjected to semi-quantitative RT-PCR with primers for Kin, GAPDH, IμF and CμR (μGLT), or IαF and CαR (αGLT) as described previously^51^,^52^.

### *Ex vivo* mouse experiments

Splenic B cells were purified from wild-type C57BL/6 mice with a negative selection mouse B cell enrichment kit (Stemcell Technologies). Cells were then cultured in complete RPMI media (Gibco) with 25 ng/mL LPS for 24 hours, followed by lentiviral transduction in the presence of 8 ng/mL polybrene (Sigma-Aldrich). Positively transduced cells were then selected with 0.6 μg/mL puromycin (Wisent Bio Products) and stimulated with 25 ng/mL recombinant mouse IL-4 (R&D Systems) for 4 days, and then analyzed by flow cytometry. Experimental protocols with mice were carried out in accordance with the University of Toronto Division of Comparative Medicine guidelines and regulations and were approved using protocol number 20011472.

### Flow cytometry assays

For CSR analysis, CH12 cells were stained with PE-conjugated anti-mouse IgA (Southern Biotech), and *ex vivo* mouse B cells were stained with PE-conjugated anti-mouse IgG1 clone A85-1 (BD Biosciences). For BrdU-PI assays, cells were incubated with 10 μM BrdU for 1 hour followed by ethanol fixation for 16 hours. Cells were then washed and incubated in 2 M HCl for 30 minutes, washed and stained with FITC-conjugated anti-BrdU clone PRB-1 (eBiosciences), and then washed and incubated in 20 μg/mL PI and 10 μg/mL RNase A for 30 minutes. For apoptosis analysis, CH12 cells were stained for Annexin V using the Annexin V-APC Apoptosis Detection Kit (eBiosciences). For mitotic index analysis, cells were CIT stimulated or irradiated and stained 24 hours post-stimulation with Alexa Fluor 488 conjugated anti-phospho-histone H3 and 20 μg/mL PI. For the above assays, cells were acquired with a FACSCalibur (BD Biosciences) and analysis performed with FlowJo VX (Tree Star Inc). For CFSE analysis, cells were pulsed with 5 μM CFSE (Celltrace, Life Technologies), washed, and CIT stimulated, as described above. Cells were collected 0, 12, 24, 36, and 48 hours following CFSE pulse and stimulation, stained, and acquired with an LSR II flow cytometer (BD Biosciences) and analyzed.

### DNA repair substrate assays

For the study of HR, NHEJ, and A-EJ respectively, CH12 were transfected via electroporation (BioRad Gene Pulser Xcell) with 5 μg of DR-GFP, pIRES-TK-GFP, or EJ2-GFP plated into 96-well plates, selected with 1 μg/mL puromycin and screened on the basis of GFP expression by flow cytometry. shGFP and shKin 24, 16, 26, 36 sequences were derived onto the pLKO_TRC005-Hygro vector and transduced into stable DR-GFP, pIRES-TK-GFP and EJ2-GFP clones. Following 2 mg/mL hygromycin selection for 6–8 days, cells were transfected with 5 μg of pCBASceI (Jasin Lab, Addgene) and GFP expression was quantified 48 hours post-transfection by flow cytometry.

### CRISPR/Cas9-mediated CSR and sequence analysis

A pair of sgRNA respectively directed to the 5′ and 3′ regions flanking Sμ and Sα were designed and cloned into px330 (Addgene #42230). To induce Cas9-mediated CSR, 5 μg each of the 5′-Sμ and 3′-Sα px330 vectors were electroporated (BioRad Gene Pulser Xcell) into AID^−/−^ CH12 cells^53^. For CSR phenotyping, cells were recovered for 3 days before staining and flow cytometry. For sequencing, genomic DNA was extracted and a 1098 base pair region spanning the switch junction was PCR amplified with Q5 polymerase (New England Biolabs) following 5 days of recovery. Junctions were cloned into the pGEM-T easy vector system (Promega) and sequenced at The Centre for Applied Genomics (Toronto).

### Switch junction sequence analysis

Switch μ-α junctions were PCR amplified with Q5 polymerase (New England Biolabs) with primers described previously^54^,^55^, and then subsequently cloned into the Zero Blunt TOPO cloning kit (Life Technologies) and sequenced at Macrogen (Seoul, South Korea) and The Centre for Applied Genomics (Toronto, Canada). Sequences with greater than two mismatches within a span of 50 base pairs of the junction were discarded. The degree of microhomology within each junction was measured permitting only one mismatch per 10 base pairs at the junction overlap.

### Switch region mutation analysis

Genomic DNA was isolated from CH12 or AID^−/−^ CH12 cells following 5 days of CIT stimulation. A 624 base pair region located 5′ of the core μ switch region was PCR-amplified with Q5 polymerase (New England Biolabs) using primers described previously^56^. The PCR product was cloned into the pGEM-T easy vector system (Promega) for sequencing analysis. Sequencing was performed at The Centre for Applied Genomics (Toronto).

### Immunofluorescence staining and confocal microscopy

Cells were irradiated with various doses of ionizing radiation (Gammacell 1000, Nordion) 1 hour prior to preparation onto poly-L-lysine slides with a cytospin centrifuge (Cyto-Tek, Sakura). Cytospin preparations were fixed with 4% PFA/PBS. Then, slides were permeabilized with 0.25% (v/v) Triton X-100/PBS and blocked with 0.1% Tween + 1% BSA/PBS for 1 h at room temperature. Slides were incubated with the following primary antibodies specific for γH2AX and secondary antibodies anti-mouse IgG1 Alexa-488 and anti-mouse Ig H + L Alexa-555 as described above. Following staining, slides were incubated with DAPI at a concentration of 0.5 μg/mL (Sigma-Aldrich) and mounted with aqueous M1289 mounting media (Sigma-Aldrich). Images were taken with a confocal microscope (Nikon, AZ-C2+) equipped with a 100x magnification oil immersion lens. Images were acquired and processed within Nikon NIS Elements software and exported for analysis with ImageJ (NIH). For foci quantification, between 50 and 100 cells were counted for number of foci in each cell per treatment per experiment.

### Statistical analysis

All analyses were performed with GraphPad Prism 6. For unpaired, two-tailed Student’s t tests, linear regression analysis, and two-way analysis of variance (ANOVA), p values of 0.05 or less were considered significant: *p < 0.05, **p < 0.01 and ***p < 0.001. All error bars represent standard deviations.

## Additional Information

**How to cite this article**: Le, M. X. *et al.* Kin17 facilitates multiple double-strand break repair pathways that govern B cell class switching. *Sci. Rep.* **6**, 37215; doi: 10.1038/srep37215 (2016).

**Publisher’s note**: Springer Nature remains neutral with regard to jurisdictional claims in published maps and institutional affiliations.

## Supplementary Material

Supplementary Information

## Figures and Tables

**Table 1:** Mutation and deletion frequency in the μ switch region.

	WT CH12		AID^−/−^ CH12	
	shGFP	shKin 24	shGFP	shKin 24
Sequences (#)	94	93	45	44
Mutations (#)	49	51	0	0
Mutation Frequency^a^	9.35 × 10^−4^	8.75 × 10^−4^	<3.56 × 10^−5^	<3.64 × 10^−5^
Mutations at G/C (%)^b^	100	100	0	0
AID hotspots mutations (%)^c^	83	78	0	0
Deletions/Insertions (#)^d^	5	17	0	0

^a^Frequency is defined as unique mutations/nucleotide sequenced.

^b^Percentage of mutations at G:C base pairs calculated over total number of mutations.

^c^Mutations at WRC and GYW motifs. W = A/T nucleotides; R = A/G nucleotides; Y = T/C nucleotides.

^d^p value = 0.0099, as measured by unpaired, two-tailed, Student’s t-test.

**Table 2:** Deletion and insertion frequency in CRISPR/Cas9-mediated CSR junctions.

	shGFP	shKin 24
Unique Sequences (#)	28	17
Deletions (#)^a^	27	16
Mean Deletion Length (nt)b,^c^	75.9	331.1
Insertions (#)^a^	2	7
Mean Insertion Length (nt)^b^	10.5	36.0

^a^Number of junction sequences harbouring deletions or insertions.

^b^Mean deletion or insertion length at the junctions as measured by number of nucleotides.

^c^p value = 0.0060, as measured by unpaired, two-tailed, Student’s t-test.
