# Supplementary material for: Kin17 facilitates multiple double-strand break repair pathways that govern B cell class switching
Source: Sci Rep. 2016 Nov 17;6:37215. doi: 10.1038/srep37215 (PMC5112545; doi:10.1038/srep37215)
Supplement: Supplementary Information [file srep37215-s1.pdf]

Kin17 facilitates multiple double-strand break repair pathways that govern B cell class switching

Michael X. Le, Dania Haddad, Alexanda K. Ling, Conglei Li, Clare C. So, Amit Chopra, Rui Hu, Jaime F. Angulo, Jason Moffat, *Alberto Martin

SUPPLEMENTARY MATERIAL

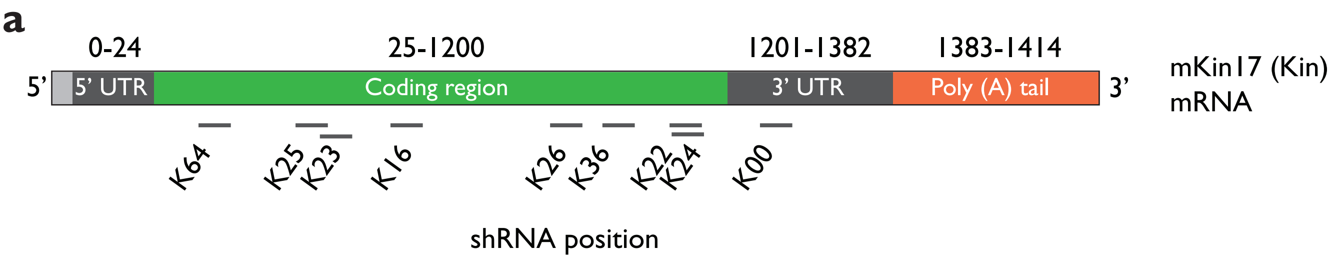

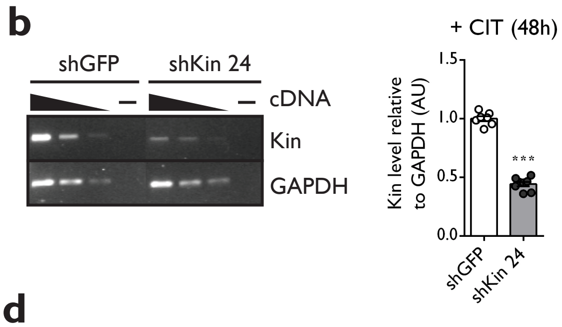

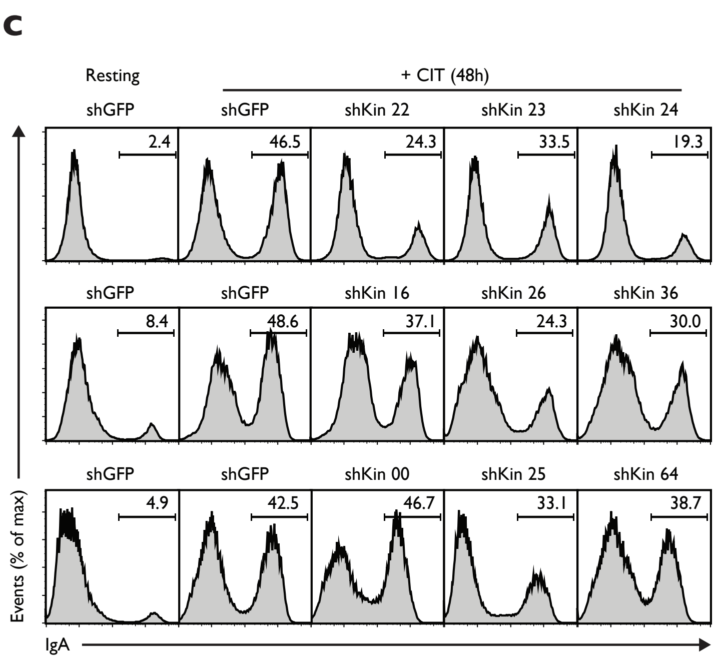

d

Experiment	shGFP (IgA+)	shKin 24 (IgA+)	Relative to shGFP
1	45.9	28.1	*61.3%*
2	45.0	19.1	*42.5%*
3	56.8	24.1	*42.5%*
4	62.5	26.0	*41.6%*
5	45.0	23.2	*51.4%*
6	55.6	21.9	*39.4%*
7	52.7	18.0	*34.1%*
8	50.3	16.8	*33.3%*
9	55.6	21.9	*39.4%*
10	43.0	18.9	*44.0%*

Supplementary Figure S1

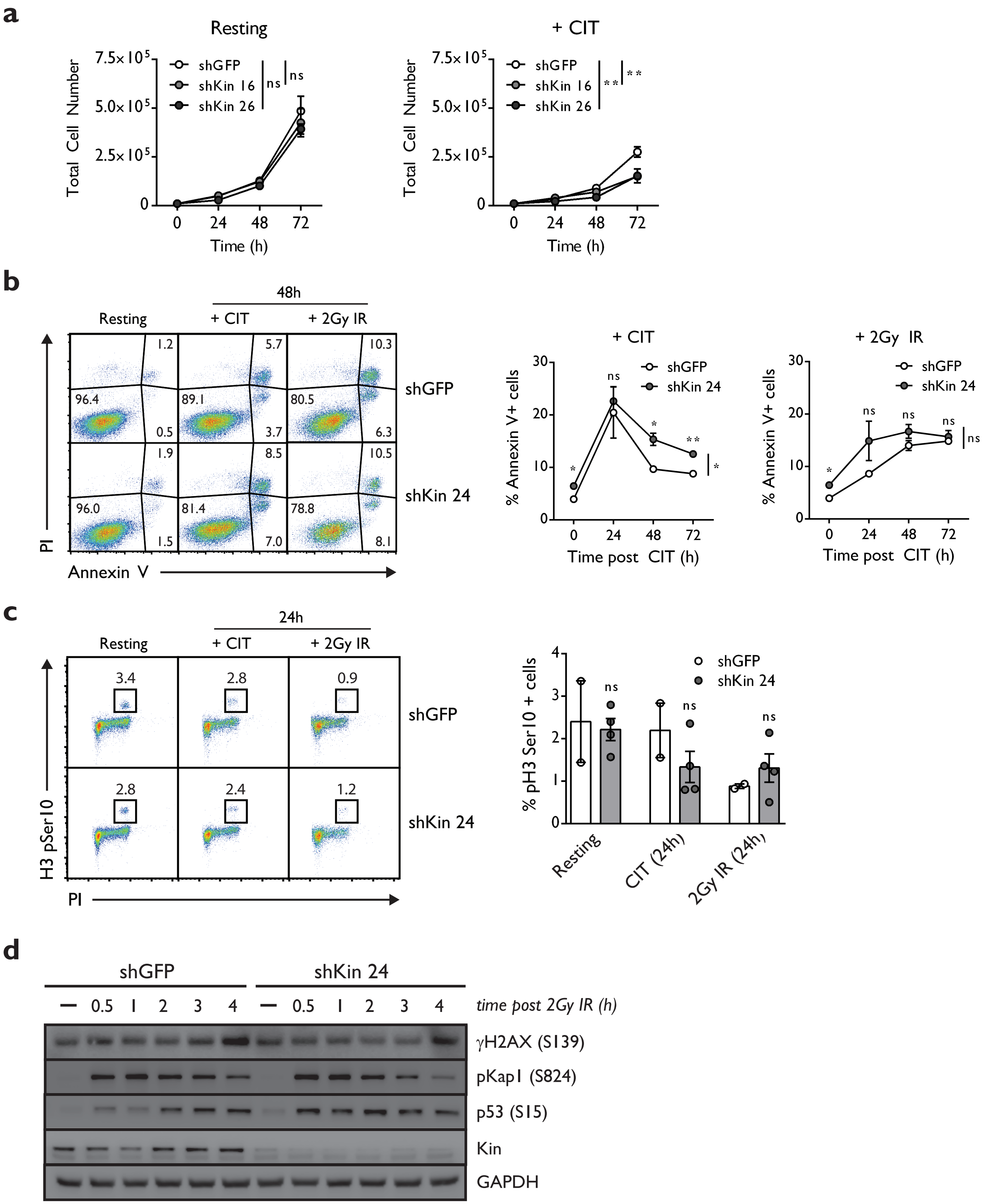

Supplementary Figure S2

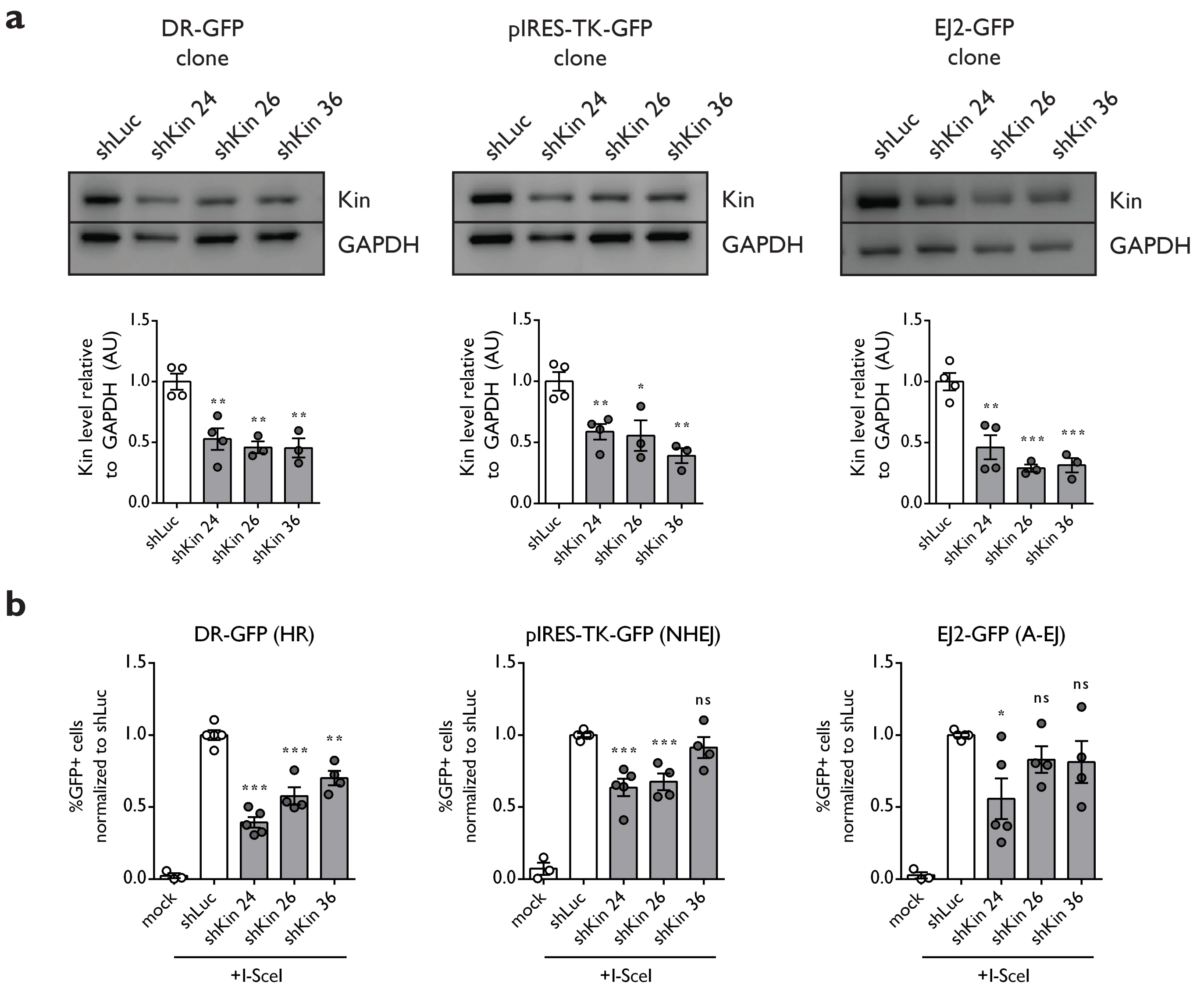

Supplementary Figure S3

SUPPLEMENTARY FIGURE LEGENDS

Supplementary Figure S1. Multiple shRNA targeting Kin reduce CSR in CH12 cells. (a) Schematic of shRNA used in this study positioned on full-length Kin mRNA, abbreviated as such: K24 = shKin24, etc. **(b)** Expression levels of Kin transcript relative to GAPDH measured by RT-PCR in CH12 cells transduced with shGFP or shKin 24. Each shRNA was assayed with 2 biological replicates in 3 independent experiments. **(c)** Surface IgA expression measured following 48 hours of CIT-stimulation in CH12 cells transduced with shGFP and the hairpins mentioned in **(a)**. **(d)** Table of 10 independent experiments measuring surface IgA expression in CH12 cells transduced with shGFP or shKin 24, highlighting the relative CSR defect in shKin 24 compared to shGFP. All experiments were analyzed by unpaired, two-tailed, Student's t-test, where applicable.

Supplementary Figure S2. Kin deficiency results in minor proliferation and apoptotic defects. (a) Growth curves of CH12 cells transduced and selected with shGFP, shKin 16, and shKin 26 while resting or CIT stimulated over a 72 hour timecourse as measured by Trypan Blue exclusion counting. Data was analyzed by two-way ANOVA. **(b)** Quantification of apoptotic cells by Annexin V/PI staining in CH12 cells transduced with shGFP or shKin 24 following 48 hours of CIT stimulation or 2 Grays of ionizing radiation. Representative plots (left) and summary graphs (right). Data was analyzed by two-way ANOVA. **(c)** Mitotic index analysis as measured by H3 phospho-Ser10 staining in CH12 cells transduced with shGFP or shKin 24 following 24 hours of CIT stimulation or 2 Grays of ionizing radiation. Representative plots (left) and summary graphs (right). Data was analyzed by two-way ANOVA. **(d)** CH12 cells transduced and selected with shGFP and shKin 24 were exposed to 2 Grays of ionizing radiation and collected at various time points post-irradiation for Western blot analysis of γH2AX (Ser139), phospho-Kap1 (Ser824), phospho-p53 (Ser 15), and Kin, all relative to GAPDH. Data is representative of 2 independent experiments.Unless otherwise indicated, all experiments were also analyzed by unpaired, two-tailed, Student's t-test.

Supplementary Figure S3. Multiple shRNA targeting Kin impair several mechanisms of DSB repair. (a) Expression levels of Kin relative to GAPDH were measured by Western blot in CH12 clones harbouring DR-GFP (left), pIRES-TK-GFP (center), and EJ2-GFP (right) transduced and selected with shRNA targeting negative control shLuc and Kin (shKin 24, 26, 36). Each shRNA was assayed with 2 biological replicates in 3 independent experiments. **(b)** CH12 clones harbouring the above substrates were transduced with negative control shLuc and shKin 24, 26, 36 and mock transfected or transfected with I-SceI expressing pCBASceI and GFP expression was quantified by flow cytometry. Each shRNA was assayed with 2 biological replicates in 3 independent experiments. All experiments were analyzed by unpaired, two-tailed, Student's t-test, where applicable.

SUPPLEMENTARY TABLES

Supplementary Table S1. List of primers used in this study.

Primer	Method	Forward Sequence	Reverse Sequence
GAPDH	Semi-quantitative RT-PCR	AACTTTGGCATTGTGGAAGG	GGAGACAACCTGGTCCTCAG
Kin	Semi-quantitative RT-PCR	CCAGCCTGCGAAGAAGAAGAA GTC	TACAAAACAAATCCCCTAACACA G
μ Germline Transcript	Semi-quantitative RT-PCR	CTCTGGCCCTGCTTATTGTTG	GAGACATTTGGGAAGGACTGACT
α Germline Transcript	Semi-quantitative RT-PCR	CCTGGCTGTTCCCCTATGAA	GAGCTGGTGGGAGTGTCAGTG
μ Switch region	Switch region mutation analysis	AATGGATACCTCAGTGGTTTTTA ATGG	GCGGCCCGGCTCATTCCAGTTCAT TACAG
μ-α Switch Junction (CIT)	Switch junction analysis	TTGAGAGCCCTAGTAAGCGAGG CTCTA	GAACTGTGAATAAGTCCAGTCAT GCTAAT
μ-α Switch Junction (CRISPR/Cas9)	Switch junction analysis	TTTGAGTACCGTTGTCTGGG	GGCACGATGAATGTGACCTG

Supplementary Table S2. List of shRNA target sequences used in this study.

Gene	shRNA	Mature Forward Target Sequence
GFP	shGFP	ACAACAGCCACAACGTCTATA
Luc	shLuc	CTTCGAAATGTCCGTTCGGTT
Kin	shKin 24	CAGAGTTGAAGGTATTCAATA
Kin	shKin 22	GCAGAGTTGAAGGTATTCAAT
Kin	shKin 23	CGTCGGCAACTGGAATTAGAA
Kin	shKin 16	GCAAGATCTGGACGATGAAGA
Kin	shKin 26	GGTCGTTAAGGAAGTGATTGA
Kin	shKin 36	GGCACTCTCGAATCCATCAAT
Kin	shKin 00	GAAACTGTGAAGCATCAAATT
Kin	shKin 25	CACCGAAAGGCTGGTACATTC
Kin	shKin 64	CTCAGCAGTTTATGGATTATT
